# Supplementary material for: Aneuploidy-induced proteostasis disruption impairs mitochondrial functions and mediates aggregation of mitochondrial precursor proteins through SQSTM1/p62
Source: Nat Commun. 2025 Jun 17;16:5328. doi: 10.1038/s41467-025-60857-4 (PMC12174339; doi:10.1038/s41467-025-60857-4)
Supplement: Supplementary file 1 — Supplementary Information [file 41467_2025_60857_MOESM1_ESM.pdf]

Aneuploidy-induced proteostasis disruption impairs mitochondrial functions and mediates aggregation of mitochondrial precursor proteins through SQSTM1/p62

Prince Saforo Amponsah[1]*, Jan-Eric Bökenkamp[1], Olha Kurpa[1], Svenja Lenhard[2], Anna Myronova[1], Daniel Osmar Vega Velazquez[1], Celina Hirschelmann[1], Christian Behrends[3], Johannes M. Herrmann[2], Markus Räschle[1], Zuzana Storchová[1]*.

[1]Molecular Genetics, Rheinland-Pfälzische Technische Universität (RPTU) Kaiserslautern-Landau, Kaiserslautern, Germany

[2]Cell Biology, Rheinland-Pfälzische Technische Universität (RPTU) Kaiserslautern-Landau, Kaiserslautern, Germany

[3]Munich Cluster for Systems Neurology (SyNergy), Ludwig-Maximilians-University München, Munich, Germany

*Correspondence: Zuzana Storchová (zuzana.storchova@rptu.de), Prince Saforo Amponsah (amponsah@rptu.de)

This file contains:

Supplementary Figures 1-10

Supplementary Tables 1-3

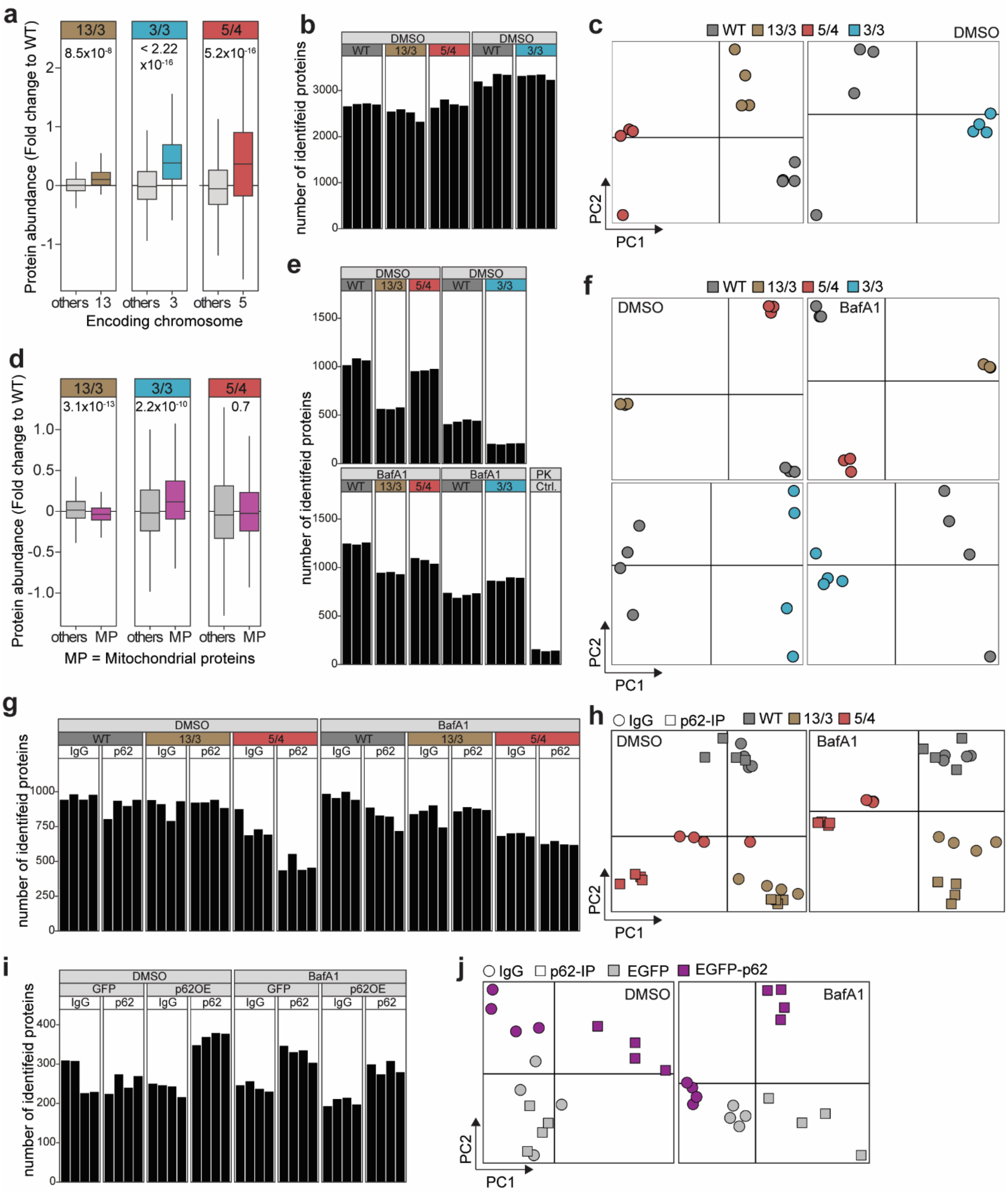
a
13/3
3/3
5/4
8.5x10-8
< 2.22 x10-16
5.2x10-16
Protein abundance (Fold change to WT)
others 13 others 3 others 5
Encoding chromosome
b
DMSO
WT 13/3 5/4
WT 3/3
number of identifeid proteins
c
WT 13/3 5/4 3/3
DMSO
PC1
PC2
d
13/3
3/3
5/4
3.1x10-13
2.2x10-10
0.7
Protein abundance (Fold change to WT)
others MP others MP others MP
MP = Mitochondrial proteins
e
DMSO
BafA1
PK Ctrl.
number of identifeid proteins
f
DMSO
BafA1
g
DMSO
BafA1
IgG p62
number of identifeid proteins
h
IgG p62-IP WT 13/3 5/4
i
DMSO
BafA1
GFP
p62OE
number of identifeid proteins
j
IgG p62-IP EGFP EGFP-p62

Supplementary Fig. 1: Expression, coverage, and principal component analyses (PCA) of proteomics data. a, Box plots of log2 fold changes in global protein abundance of polysomic cells relative to parental the HCT116 cells. Boxplots represent median with 25th and 75th percentile. Whiskers extend to the largest and smallest values, respectively, no further than 1.5x inter-quartile range from the respective bound (Tukey method). Changes were compared between proteins that are encoded from the extra chromosomes and all other proteins detected by mass spectrometry. *P*-values are derived from two-sided Wilcoxon's rank sum tests. **b,** Number of proteins from cytosolic p62 proximal proteome of DMSO treated cells. **c,** PCA of proteomics data from (**b**). **d,** Box plots of log2 fold changes in mitochondrial protein abundance of polysomic cells relative to the parental HCT116 cells. Boxplots represent median with 25th and 75th percentile. Whiskers extend to the largest and smallest values, respectively, no further than 1.5x inter-quartile range from the respective bound (Tukey method). Changes were compared between MitoCarta3.0 proteins and all other proteins detected by mass spectrometry. *P*-values are derived from two-sided Wilcoxon's rank sum tests. **e,** Number of proteins from autophagosome lumen p62-proximal proteome of DMSO and Bafilomycin A1 treated cells. **f,** PCA of proteomics data from (**d**). PK Ctrl. = proteinase K resistant control (clarified lysates treated with both proteinase K and RAPIGest to identify proteins not digested by proteinase K). **g,** Number of proteins from proteomics data of IP-MS in DMSO and Bafilomycin A1 treated WT, 13/3 and 5/4 cells. **h,** PCA of proteomics data from (**g**). **i,** Number of proteins from proteomics data of IP-MS in DMSO and Bafilomycin A1 treated WT cells transiently transfected with EGFP and EGFP-p62 overexpression plasmids. **j,** PCA of proteomics data from (**i**).

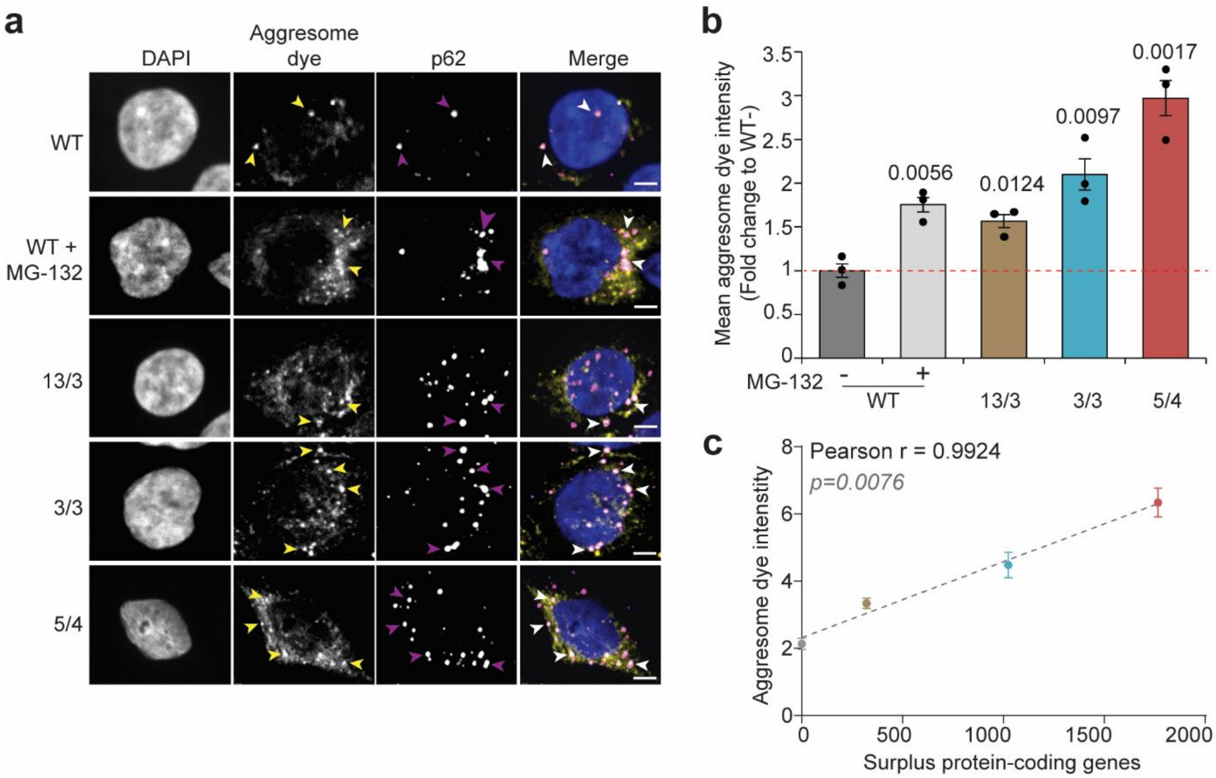

Supplementary Fig. 2: Protein aggregation is increased in polysomic cells. a, Representative confocal images of aggresome dye fluorescence (yellow) and p62 immunofluorescence (magenta) in WT, 13/3, 3/3 and 5/4 cells. WT cells treated with the proteasomal inhibitor MG-132 were used as positive control. DAPI (blue) is used as a nuclear stain. Images are collapsed from Z-stacks. Scale bar is 10 µm. White arrow heads indicate p62-aggresome colocalization. **b,** Quantification of the aggresome dye fluorescence intensity from (**a**). Data is shown as mean ± s.e.m. fold change to untreated WT cells from n = 3 independent experiments, and individual replicates are plotted as dots. P-values represent two-tailed unpaired Student's t-test. **c,** Correlation of the mean ± s.e.m. aggresome dye intensity from n = 3 independent experiments with the number of surplus protein-coding genes in the cell lines used. Two-tailed P-value is indicated.

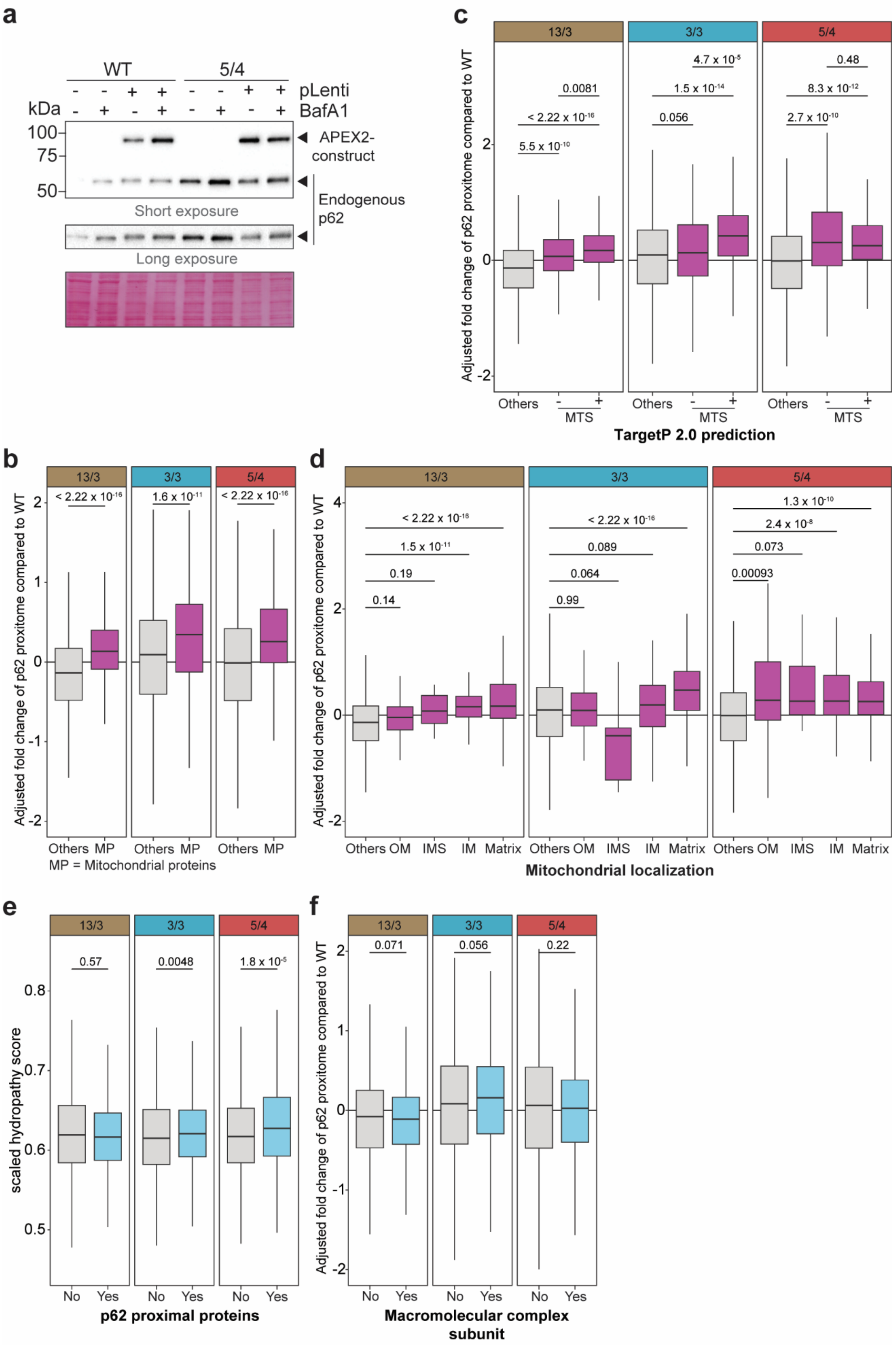
a
WT
5/4
pLenti
BafA1
kDa
100
75
50
APEX2-construct
Endogenous p62
Short exposure
Long exposure
b
13/3
3/3
5/4
< 2.22 x 10-16
1.6 x 10-11
< 2.22 x 10-16
Adjusted fold change of p62 proximitome compared to WT
Others MP
MP = Mitochondrial proteins
c
13/3
3/3
5/4
0.0081
< 2.22 x 10-16
5.5 x 10-10
4.7 x 10-5
1.5 x 10-14
0.056
0.48
8.3 x 10-12
2.7 x 10-10
Adjusted fold change of p62 proximitome compared to WT
Others
MTS
TargetP 2.0 prediction
d
13/3
3/3
5/4
< 2.22 x 10-16
1.5 x 10-11
0.19
0.14
< 2.22 x 10-16
0.089
0.064
0.99
1.3 x 10-10
2.4 x 10-8
0.073
0.00093
Adjusted fold change of p62 proximitome compared to WT
Others OM IMS IM Matrix
Mitochondrial localization
e
13/3
3/3
5/4
0.57
0.0048
1.8 x 10-5
scaled hydropathy score
No Yes
p62 proximal proteins
f
13/3
3/3
5/4
0.071
0.056
0.22
Adjusted fold change of p62 proximitome compared to WT
No Yes
Macromolecular complex subunit

Supplementary Fig. 3: Characterization of p62 proximal proteins in polysomic cells. a, Immunoblot showing stable expression of the APEX2-p62 construct in WT and 5/4 cells in the absence and presence of BafA1. The expression of endogenous p62 was also monitored. Ponceau staining is used as loading control. Boxplots indicating **b,** adjusted fold changes in abundance of mitochondrial proteins (magenta) compared to all other measured proteins (grey) in 13/3, 3/3 and 5/4 polysomic cells relative to diploid parental cells (WT), **c,** distribution of the enriched p62-proximal mitochondrial proteins in (**b**) based on whether they possess canonical mitochondrial targeting sequences (MTS) according to TargetP 2.0 prediction, **d,** fold change distribution of the p62-proximal mitochondrial proteins in (**b**) according to subcellular localization. **e,** Hydrophobicity score, and **f,** Identity as subunit of macromolecular complexes (CORUM) of all enriched p62-proximal proteins (blue) in 13/3, 3/3 and 5/4 polysomic cells relative to diploid parental cells (WT). Boxplots represent median with 25th and 75th percentile. Whiskers extend to the largest and smallest values, respectively, no further than 1.5x inter-quartile range from the respective bound (Tukey method). *P*-values are derived from two-sided Wilcoxon's ranks sum tests.

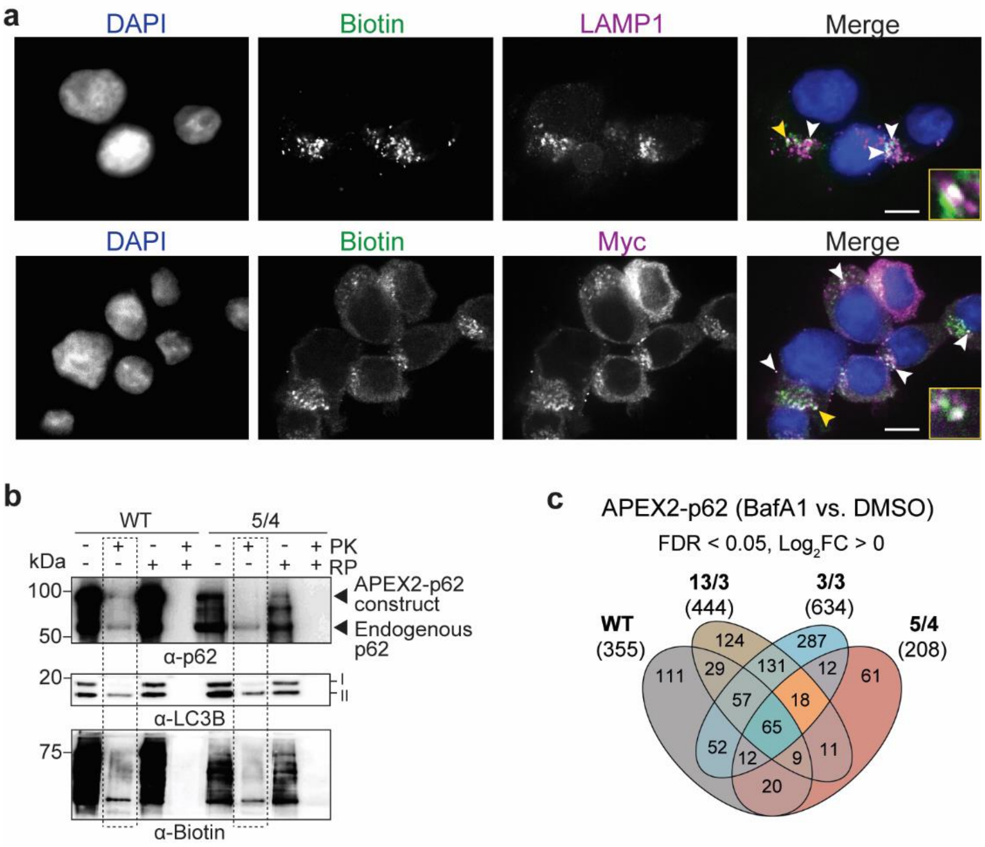

Supplementary Fig. 4: Analyses of autophagosome luminal p62 proximal cargo. a, Representative confocal images indicating separate colocalizations of biotin (green) with APEX2-p62 (magenta) and the autolysosomal marker LAMP1 (magenta). DAPI (blue) is used as a nuclear stain. Images are collapsed from Z-stacks. Scale bar is 10 µm. Colocalization events are indicated by white and yellow arrowheads. Insets represent colocalizations indicated by yellow arrowheads. **b,** Immunoblot of the protease protection assay. Cell homogenates were incubated with Proteinase K (PK), RAPIGest (RP), or both and blotted for p62, LC3B and biotin. Dotted boxes show the condition analyzed by mass spectrometry. The presence of only LC3B-II in this condition confirms successful enrichment of protease-protected autophagosome lumen cargo. **c,** Venn diagram showing the distribution of the number of autophagosomal lumen p62 cargo candidates between karyotypes (related to **Fig. 3b, c**).

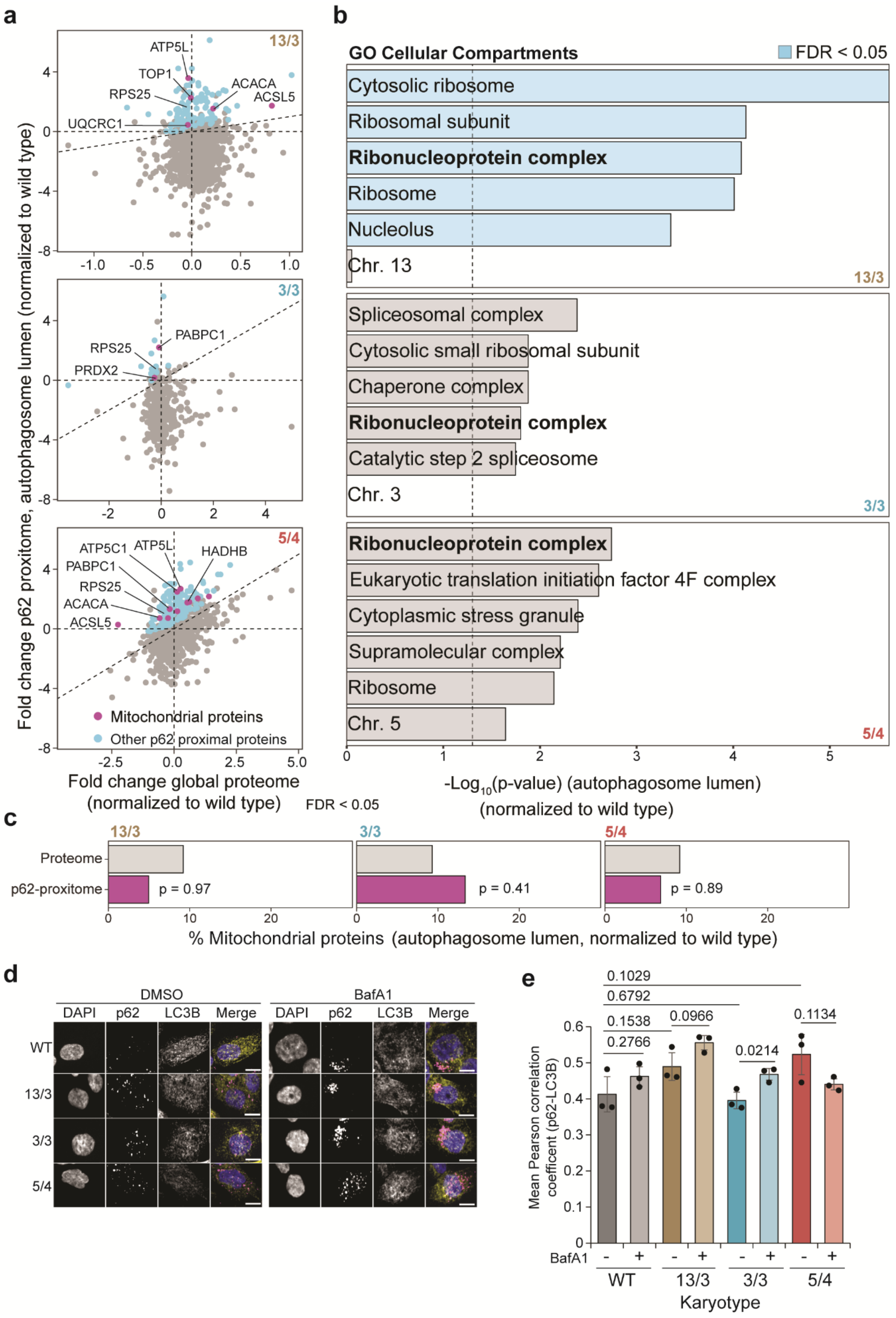

a
13/3
ATP5L
TOP1
RPS25
ACACA
ACSL5
UQCRC1
3/3
PABPC1
RPS25
PRDX2
5/4
ATP5C1
ATP5L
HADHB
PABPC1
RPS25
ACACA
ACSL5
Mitochondrial proteins
Other p62 proximal proteins
Fold change p62 proximome, autophagosome lumen (normalized to wild type)
Fold change global proteome (normalized to wild type)
b
GO Cellular Compartments
FDR < 0.05
Cytosolic ribosome
Ribosomal subunit
Ribonucleoprotein complex
Ribosome
Nucleolus
Chr. 13
13/3
Spliceosomal complex
Cytosolic small ribosomal subunit
Chaperone complex
Ribonucleoprotein complex
Catalytic step 2 spliceosome
Chr. 3
3/3
Ribonucleoprotein complex
Eukaryotic translation initiation factor 4F complex
Cytoplasmic stress granule
Supramolecular complex
Ribosome
Chr. 5
5/4
-Log10(p-value) (autophagosome lumen) (normalized to wild type)
FDR < 0.05
c
13/3
3/3
5/4
Proteome
p62-proximotome
p = 0.97
p = 0.41
p = 0.89
% Mitochondrial proteins (autophagosome lumen, normalized to wild type)
d
DMSO
BafA1
DAPI
p62
LC3B
Merge
WT
13/3
3/3
5/4
e
0.1029
0.6792
0.1538
0.0966
0.2766
0.0214
0.1134
Mean Pearson correlation coefficent (p62-LC3B)
BafA1
WT
13/3
3/3
5/4
Karyotype

Supplementary Fig. 5: Autophagosome content profiling reveals ribosome and ribonucleoproteins as predominant p62 proximal proteins in autophagosome lumen of polysomic cells. **a**, Scatter plot of the fold changes in global protein abundance in untreated 13/3, 3/3 and 5/4 polysomic cell lines relative to the parental cell line (WT) and the corresponding changes in abundance of the autophagosome lumen p62-proximal proteins relative to WT. Colored dots represent proteins with significantly higher abundance changes of autophagosome lumen p62-proximal proteins in polysomic cells (FDR < 0.05). **b**, Gene ontology (GO) over-representation analysis of the enriched p62-proximal proteins in (**a**). Blue bars represent negative log-transformed *p*-values (one-sided hypergeometric test) for GO terms with FDR < 0.05. **c**, Percentage of mitochondrial proteins in the measured lumen proteome (grey) and the corresponding percentage within the proteins increased in the autophagosome lumen p62 proximal proteome (magenta) of 13/3, 3/3 and 5/4 cells relative to WT. *P*-values represent results of one-sided hypergeometric test, evaluating the differences in representation of mitochondrial proteins. **d**, Representative confocal images of p62 (magenta) and LC3B (yellow) immunofluorescence in WT, 13/3, 3/3 and 5/4 cells treated without or with BafA1. DAPI (blue) is used as a nuclear stain. Images are collapsed from Z-stacks. Scale bar is 10 µm. **e,** Quantification of the mean Pearson correlation coefficient for p62-LC3B colocalization from (**d**). Data is shown as mean ± s.d. from n = 3 independent experiments. *P*-values represent two-tailed unpaired Student’s t-test.

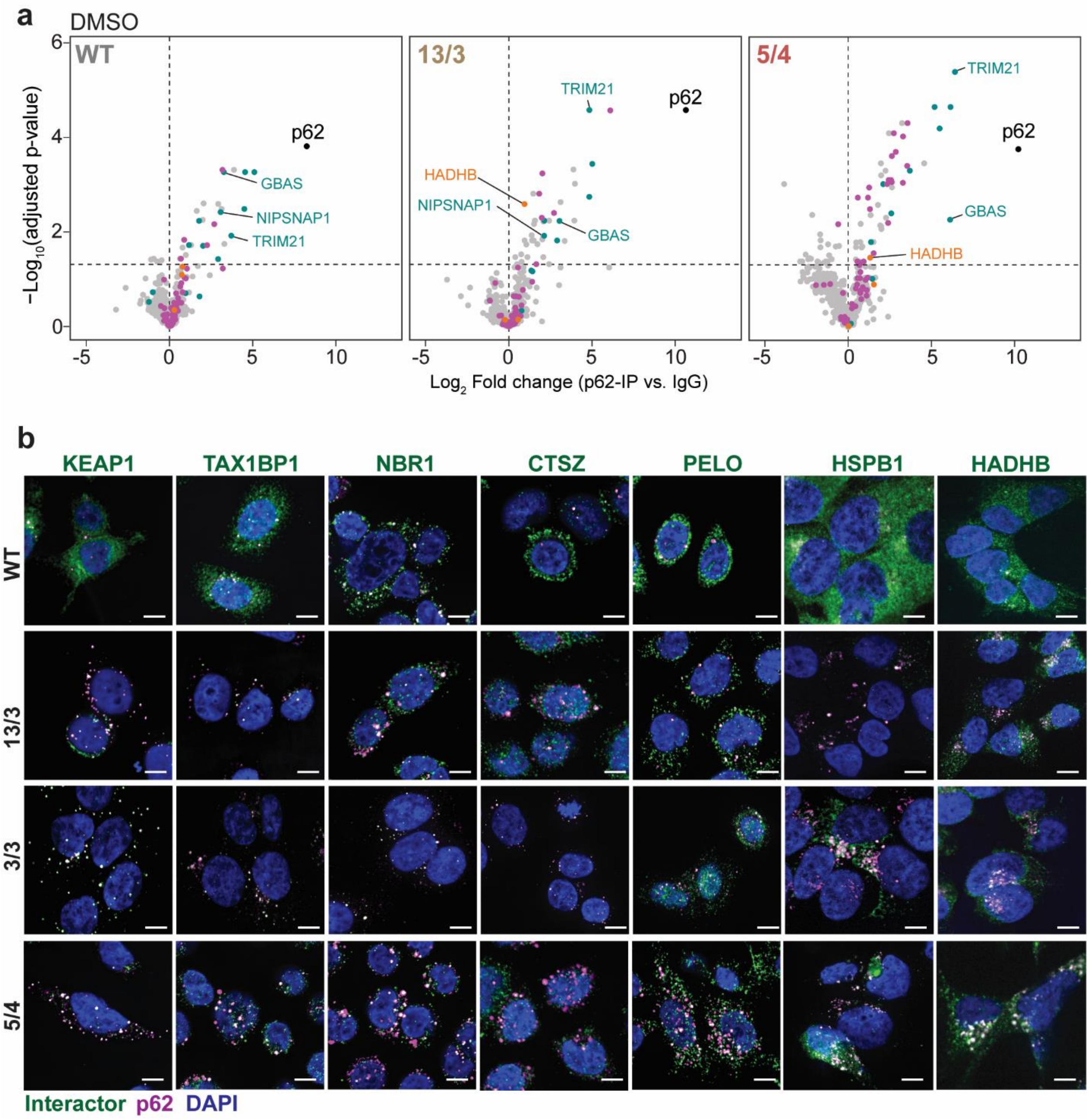

Supplementary Fig. 6: Confirmation of p62 interactors. a, Volcano plots showing log_2-transformed fold change of enriched p62 interactors in DMSO (vehicle) treated WT, 13/3 and 5/4 cells highlighting mitochondrial (magenta), karyotype-independent (cyan) and polysomic-specific (orange) interactors. **b,** Representative confocal images showing colocalization of p62 (magenta) with selected interactors (green) in WT, 13/3, 3/3 and 5/4 cells (related to **Fig. 4b**). DAPI (blue) is used as nuclear stain. Images are collapsed from Z-stacks. Scale bar is 10 µm.

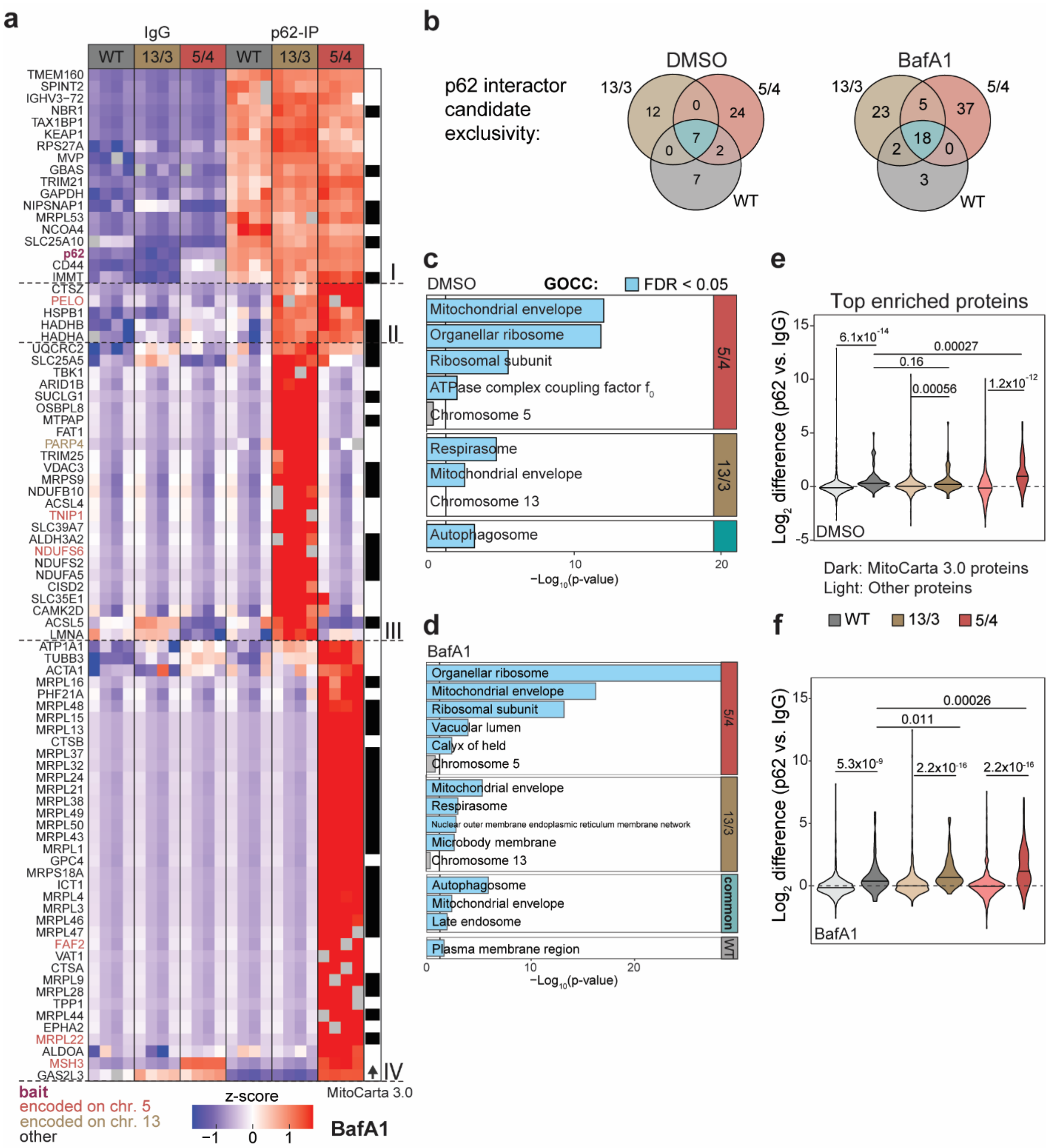
a
IgG
p62-IP
WT
13/3
5/4
WT
13/3
5/4
TMEM160
SPINT2
IGHV3−72
NBR1
TAX1BP1
KEAP1
RPS27A
MVP
GBAS
TRIM21
GAPDH
NIPSNAP1
MRPL53
NCOA4
SLC25A10
p62
CD44
IMMT
CTSZ
PELO
HSPB1
HADHB
HADHA
UQCRC2
SLC25A5
TBK1
ARID1B
SUCLG1
OSBPL8
MTPAP
FAT1
PARP4
TRIM25
VDAC3
MRPS9
NDUFB10
ACSL4
TNIP1
SLC39A7
ALDH3A2
NDUFS6
NDUFS2
NDUFA5
CISD2
SLC35E1
CAMK2D
ACSL5
LMNA
ATP1A1
TUBB3
ACTA1
MRPL16
PHF21A
MRPL48
MRPL15
MRPL13
CTSB
MRPL37
MRPL32
MRPL24
MRPL21
MRPL38
MRPL49
MRPL50
MRPL43
MRPL1
GPC4
MRPS18A
ICT1
MRPL4
MRPL3
MRPL46
MRPL47
FAF2
VAT1
CTSA
MRPL9
MRPL28
TPP1
MRPL44
EPHA2
MRPL22
ALDOA
MSH3
GAS2L3
I
II
III
IV
bait
encoded on chr. 5
encoded on chr. 13
other
z-score
−1
0
1
MitoCarta 3.0
BafA1
b
p62 interactor candidate exclusivity:
DMSO
13/3
5/4
WT
12
0
24
7
0
2
7
BafA1
13/3
5/4
WT
23
5
37
18
2
0
3
c
DMSO
GOCC:
FDR < 0.05
Mitochondrial envelope
Organellar ribosome
Ribosomal subunit
ATPase complex coupling factor f_0
Chromosome 5
5/4
Respirasome
Mitochondrial envelope
Chromosome 13
13/3
Autophagosome
0
10
20
$-Log_{10}$(p-value)
d
BafA1
Organellar ribosome
Mitochondrial envelope
Ribosomal subunit
Vacuolar lumen
Calyx of held
Chromosome 5
5/4
Mitochondrial envelope
Respirasome
Nuclear outer membrane endoplasmic reticulum membrane network
Microbody membrane
Chromosome 13
13/3
Autophagosome
Mitochondrial envelope
Late endosome
common
Plasma membrane region
WT
0
10
20
$-Log_{10}$(p-value)
e
Top enriched proteins
Log_2 difference (p62 vs. IgG)
15
10
5
0
-5
$6.1x10^{-14}$
0.00027
0.16
0.00056
$1.2x10^{-12}$
DMSO
Dark: MitoCarta 3.0 proteins
Light: Other proteins
f
WT
13/3
5/4
Log_2 difference (p62 vs. IgG)
15
10
5
0
0.00026
0.011
$5.3x10^{-9}$
$2.2x10^{-16}$
$2.2x10^{-16}$
BafA1

Supplementary Fig. 7: Analyses of p62 interactome by immunoprecipitation mass spectrometry. a, Hierarchical clustering of enriched p62 interactors from (**Fig. 4b**) according to their z-scores. Black boxes represent mitochondrial proteins as defined by the MitoCarta3.0 inventory, clusters I – IV represent proteins with similar pattern of enrichment. Missing values from quadruplicate experiments are indicated in grey. **b**, Venn diagram showing distribution of enriched p62 interactors among karyotypes when compared to IgG control from all groups, in DMSO and BafA1-treated cells. Gene ontology (GO) over-representation analysis of enriched p62 interactors in **c**, DMSO, and **d**, BafA1-treated cells. Blue bars represent negative log-transformed *p*-values (one-sided hypergeometric test) for GO terms with FDR < 0.05. Representatives from clusters of related GO terms are shown (see Methods). Comparison of fold differences in enrichment of mitochondrial proteins, as defined by the MitoCarta3.0 inventory (dark colors), to all other proteins in the measured p62 interactome (light colors) in **e,** DMSO, and **f,** BafA1-treated cells. *P*-values are derived from two-sided Wilcoxon's ranks sum tests.

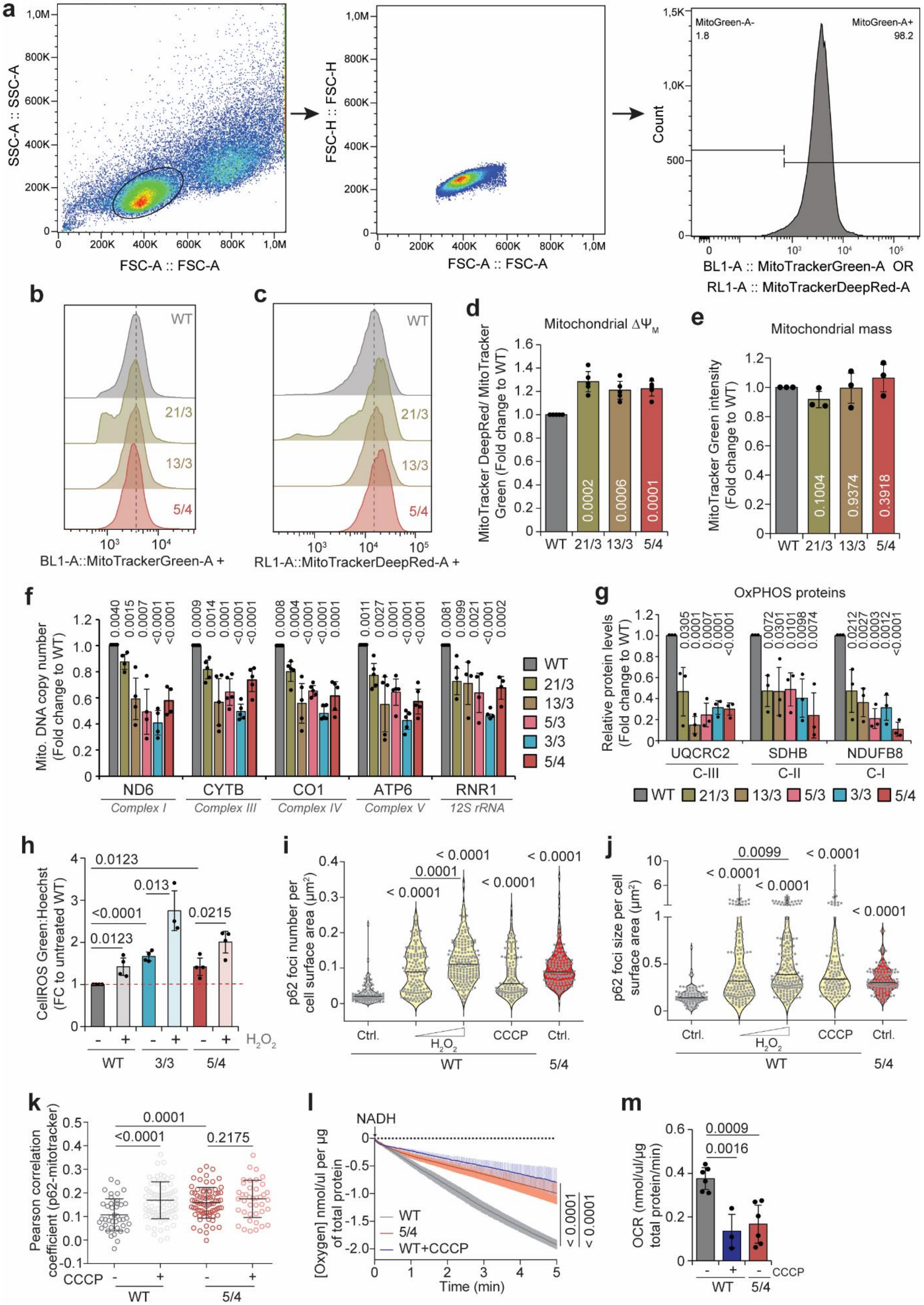

a
SSC-A :: SSC-A
FSC-A :: FSC-A
FSC-H :: FSC-H
FSC-A :: FSC-A
MitoGreen-A-
1.8
MitoGreen-A+
98.2
Count
BL1-A :: MitoTrackerGreen-A OR
RL1-A :: MitoTrackerDeepRed-A
b
WT
21/3
13/3
5/4
BL1-A::MitoTrackerGreen-A +
c
WT
21/3
13/3
5/4
RL1-A::MitoTrackerDeepRed-A +
d
Mitochondrial ΔΨM
MitoTracker DeepRed/ MitoTracker Green (Fold change to WT)
0.0002
0.0006
0.0001
WT 21/3 13/3 5/4
e
Mitochondrial mass
MitoTracker Green intensity (Fold change to WT)
0.1004
0.9374
0.3918
WT 21/3 13/3 5/4
f
Mito. DNA copy number (Fold change to WT)
0.0040 0.0015 0.0007 <0.0001 <0.0001
0.0009 0.0014 0.0001 <0.0001 <0.0001
0.0008 0.0004 <0.0001 <0.0001 0.0001
0.0011 0.0027 0.0001 <0.0001 <0.0001
0.0081 0.0099 0.0021 <0.0001 0.0002
ND6 Complex I
CYTB Complex III
CO1 Complex IV
ATP6 Complex V
RNR1 12S rRNA
WT 21/3 13/3 5/3 3/3 5/4
g
OxPHOS proteins
Relative protein levels (Fold change to WT)
0.0305 0.0007 0.0007 0.0001 <0.0001
0.0072 0.0301 0.0101 0.0098 0.0074
0.0212 0.0027 0.0003 0.0012 <0.0001
UQCRC2 C-III
SDHB C-II
NDUFB8 C-I
WT 21/3 13/3 5/3 3/3 5/4
h
CellROS Green:Hoechst (FC to untreated WT)
0.0123
0.013
<0.0001
0.0215
0.0123
H2O2
WT 3/3 5/4
i
p62 foci number per cell surface area (µm²)
< 0.0001
0.0001
< 0.0001
< 0.0001
< 0.0001
Ctrl. H2O2 CCCP Ctrl.
WT 5/4
j
p62 foci size per cell surface area (µm²)
0.0099
< 0.0001
< 0.0001
< 0.0001
< 0.0001
< 0.0001
Ctrl. H2O2 CCCP Ctrl.
WT 5/4
k
Pearson correlation coefficient (p62-mitotracker)
0.0001
<0.0001
0.2175
CCCP
WT 5/4
l
NADH
[Oxygen] nmol/ul per µg of total protein
WT
5/4
WT+CCCP
< 0.0001
< 0.0001
Time (min)
m
OCR (nmol/ul/µg total protein/min)
0.0009
0.0016
CCCP
WT 5/4

Supplementary Fig. 8: Analyses of mitochondrial functions and reactive oxygen species (ROS) production. a, Gating strategy for flow cytometry. Representative histograms of **b,** Mitotracker Green FM and **c,** Mitotracker DeepRed FM fluorescence measured by flow cytometry. **d,** Mitochondrial membrane potential quantified as ratio of Mitotracker DeepRed FM to Mitotracker Green FM intensity. Mean ± s.d. fold change to WT from *n* = 5 independent experiments are shown. **e,** Mitochondrial mass determined from Mitotracker Green FM intensities. Mean ± s.d. fold change to WT from *n* = 3 independent experiments are shown, individual replicates are plotted as dots. *P*-values represent two-tailed unpaired Student's *t*-test. **f**, Relative mitochondrial DNA (mt-DNA) copy number determined by qPCR of mitochondrially encoded respiratory chain complex subunits, normalized to the nuclear $ß_2$-microglobulin gene (see **Fig. 5g**). Mean ± s.d. fold change to WT from *n* = 4-5 independent experiments are shown, individual replicates are plotted as dots. *P*-values represent two-tailed unpaired Student's *t*-test. **g**, Quantification of expression levels of selected mitochondrial proteins from (**Fig. 5h**). Mean ± s.d. fold change to WT from *n* = 3 independent experiments are shown, individual replicates are plotted as dots. *P*-values represent two-tailed unpaired Student's *t*-test. **h,** Quantification of cellular ROS levels in the WT, 3/3 and 5/4 cells. Hydrogen peroxide (H_2O_2) treatment was used as positive control to induce ROS production. Mean ± s.d. fold change to untreated WT from *n* = 3-4 independent experiments is shown, individual replicates are plotted as dots. *P*-values represent two-tailed unpaired Student's *t*-test. Quantification of **i,** p62 foci number per cell surface area in μm^2, and **j**, p62 foci size per cell surface area in μm^2 in diploid parental cells treated without (Ctrl) or with 0.4 mM H_2O_2, 1 mM H_2O_2 or 10 µM CCCP for 1 h, as well as polysomic 5/4 cells. Violin plots indicate mean and distribution of data, while dots represent individual cells from *n* = 3 independent experiments. *P*-values represent non-parametric ANOVA (Kruskal–Wallis statistic for (**i**) 160.8, $p < 0.0001$; for (**j**) 127.5, $p < 0.0001$) followed by Dunn's multiple comparisons test. **k,** Quantification of the Pearson correlation coefficient for p62-mitochondria colocalization in WT and 5/4 cells without and with 10 µM CCCP treatment for 1 h. Individual data of cells from *n* = 3 independent experiments and mean are shown. Error bars represent standard deviation. *P*-values represent one-way ANOVA followed by Sidak's multiple comparisons test. **l,** NADH-induced oxygen consumption of mitochondria isolated from WT and 5/4 cells. In a control reaction, isolated mitochondria from WT cells were treated with 10 mM CCCP for 15 min prior to oxygen consumption measurement. Data is mean ± s.e.m. of *n* = 3-6 independent assays. *P*-value represent one-tailed paired *t*-test. **m,** Oxygen consumption rate (OCR) determined from (**l**). *P*-values represent two-tailed unpaired Student's *t*-test.

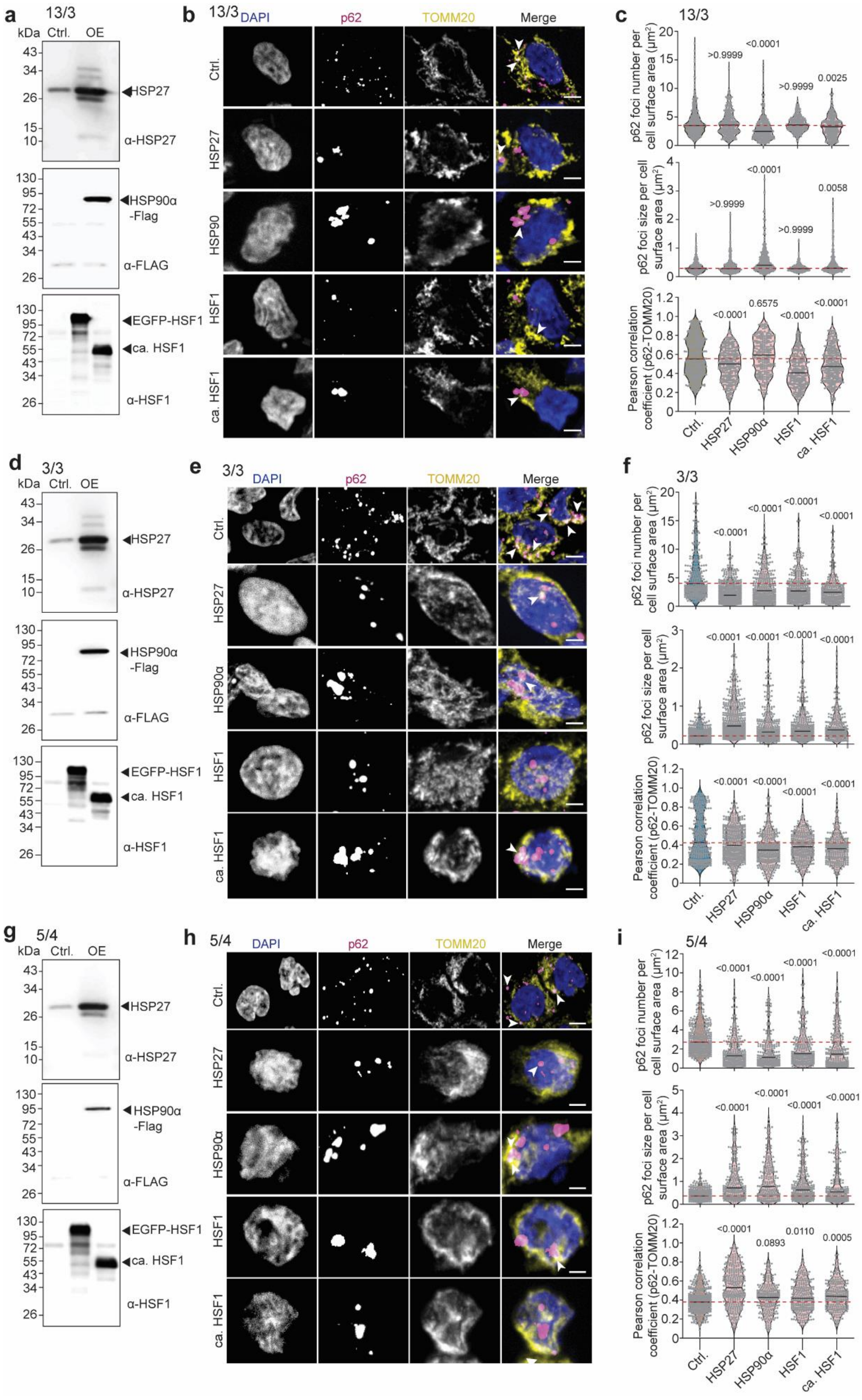

a
13/3
kDa Ctrl. OE
HSP27
α-HSP27
HSP90α -Flag
α-FLAG
EGFP-HSF1
ca. HSF1
α-HSF1
b
DAPI p62 TOMM20 Merge
Ctrl.
HSP27
HSP90
HSF1
ca. HSF1
c
p62 foci number per cell surface area (µm²)
p62 foci size per cell surface area (µm²)
Pearson correlation coefficient (p62-TOMM20)
>0.9999
<0.0001
0.0025
0.0058
0.6575
d
3/3
e
f
HSP90α
g
5/4
h
i
0.0893
0.0110
0.0005

Supplementary Fig. 9: Transient overexpression of heat shock factors in polysomic cells. **a, d, g,** Representative immunoblot showing overexpression of HSP27, HSP90α-Flag, EGFP-HSF1 and constitutively active HSF1 in HCT116 13/3 (**a**), 3/3 (**d**) and 5/4 (**g**) cell lines. Mock-transfected cells (Ctrl.) are used as control. **b, e, h,** Representative confocal images of p62 foci (magenta) and TOMM20 (yellow) in the 13/3 (**b**), 3/3 (**e**) and 5/4 (**h**) cell lines overexpressing heat shock proteins. DAPI (blue) is used as nuclear stain. Images are collapsed from Z-stacks. Scale bar is 10 µm. **c, f, i,** Quantifications of p62 foci number per cell surface area in μm^2 (top), foci size per cell surface area in μm^2 (middle) and the Pearson correlation coefficient (PCC) for p62-TOMM20 colocalization (bottom) in the samples from (**b, e, h**). Violin plots indicate mean and distribution of data while dots represent individual cells from n = 3 independent experiments. *P*-values for p62 foci represent non-parametric ANOVA in **c:** (Kruskal–Wallis statistic for (foci number) 86.95, $p < 0.0001$; for (foci size) 86.51, $p < 0.0001$), **f:** (Kruskal–Wallis statistic for (foci number) 145.5, $p < 0.0001$; for (foci size) 197.9, $p < 0.0001$), **i:** (Kruskal–Wallis statistic for (foci number) 195.4, $p < 0.0001$; for (foci size) 137.3, $p < 0.0001$), followed by Dunn's multiple comparisons test. *P*-values for PCC represent one-way ANOVA followed by Sidak's multiple comparisons test.

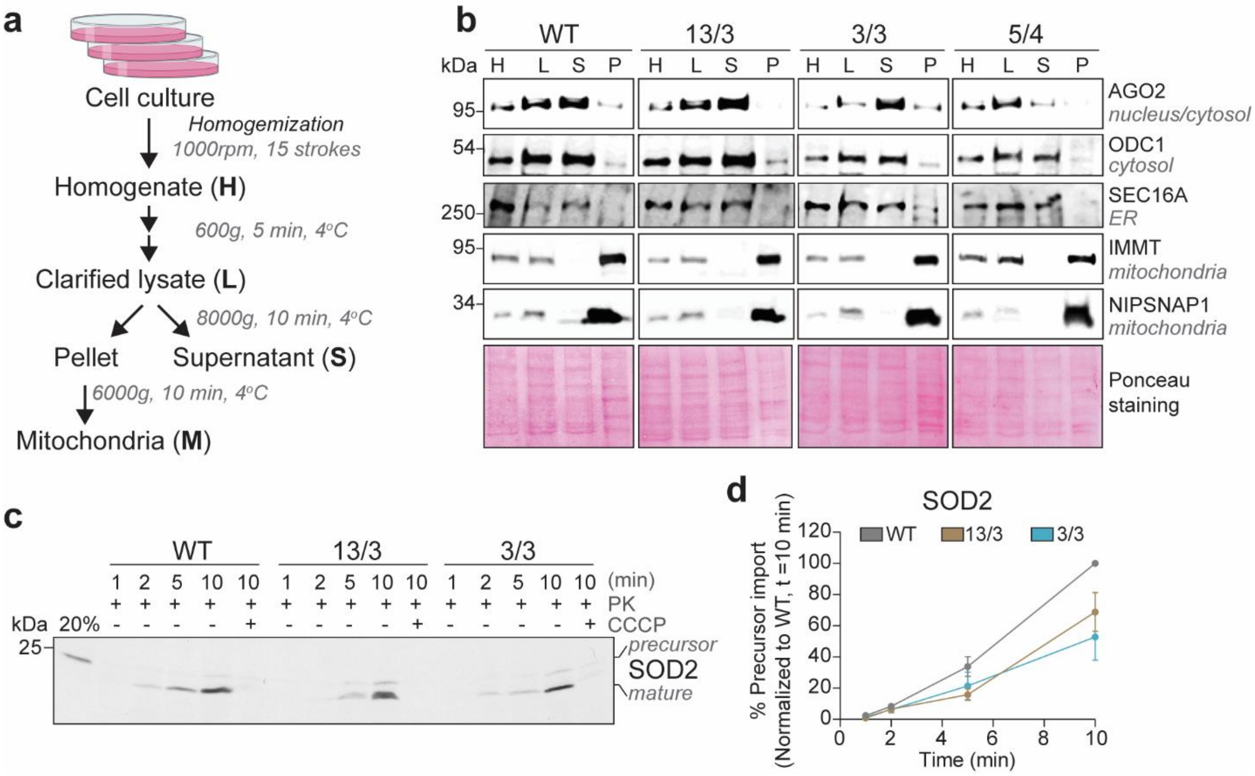

Supplementary Fig. 10: Protein import into isolated mitochondria. a, Schematic representation of procedure for mitochondrial isolation from human cells by differential centrifugation. **b,** Representative immunoblots to control for mitochondrial isolation in (**a**) from all the cell lines used. **c,** Representative image and **d,** Quantification of import kinetic of synthesized ^{35}S-methonine-labeled human SOD2 into mitochondria isolated from WT, 13/3, and 3/3 cells. The SOD2 precursor is processed upon reaching the matrix (i.e., mature form). CCCP depletes mitochondrial membrane potential thereby preventing precursor protein import. 20 % of the synthesized substrate (precursor) is loaded for comparison. All samples are resolved by SDS-PAGE and visualized by autoradiography. Data represents mean ± s.e.m. of *n* = 3 independent import assays. The data is normalized to WT, t = 10 mins (set to 100 %).

Supplementary Table 1: List of plasmids

**Name**	**Purpose**	**Source**
pHDM-Hgpm2	Lentivirus packaging	Brass et al. 2008
pHDM-tat1b	Lentivirus packaging	Brass et al. 2008
pHDM-VSV-G	Lentivirus packaging	Brass et al. 2008
pRC-CMV-rev1b	Lentivirus packaging	Brass et al. 2008
pHAGE-myc-APEX2-p62	Stable transfection	Zellner et al. 2021
pCDNA3.1 HSP90α-Flag	Transient transfection	Kind gift from Len Neckers
pCDNA3.1+ ca. HSF1	Transient transfection	Kind gift from Ulrich Hartl
pEGFP-N2 HSF1	Transient transfection	Kind gift from Ulrich Hartl
pCMV5 HSP27	Transient transfection	Kind gift from Bianca Brundel
pEGFP-N3	Transient transfection	Kind gift from Anne Simonson
pEGFP-p62	Transient transfection	Lamark et al. 2003

Supplementary Table 2: List of antibodies

Product	Species	Supplier	Product number
Primary antibodies:			
Anti p62 (SQSTM-1) Ick ligand	Mouse	BD transduction	610833/610832
Anti p62/ SQSTM1 (C-terminus)	Guinea pig	Progen	GP62-C
Anti p62	Mouse	Santa Cruz	sc-28359
Anti LC3B	Rabbit	Abcam	ab48394
Anti BIOTIN	Rabbit	Abcam	ab1227
Anti c-MYC	Goat	Bethyl Labs	A190-104A
Anti LAMP1 [H4A3]	Mouse	Abcam	ab25630
Anti KEAP1	Mouse	Santa Cruz	sc-365626
Anti TAX1BP1	Mouse	Santa Cruz	sc-393143
Anti NBR1	Mouse	Santa Cruz	sc-130380
Anti CTSZ	Mouse	Santa Cruz	sc-376976
Anti PELO	Mouse	Santa Cruz	sc-393418
Anti HSP27	Mouse	Enzo Life Sciences	ADI-SPA-800-D
Anti HSF1	Rabbit	Santa Cruz	sc-9144
Anti FLAG	Rabbit	Sigma-Aldrich	F7425
Anti GFP	Mouse	Santa Cruz	sc-9996
Anti GFP	Rat	Chromotek	3h9-100
Anti IMMT/Mitofilin	Rabbit	Biomol	A305-023A-M
Anit MRPL45 (E-12)	Mouse	Santa Cruz	sc-515563
Anti TOMM20 [EPR15581-54]	Rabbit	Abcam	ab186735
Anti HADHA (E-8)	Mouse	Santa Cruz	sc-374497
Anti HADHB (E-1)	Mouse	Santa Cruz	sc-271495
Total OXPHOS Rodent WB Antibody Cocktail	Mouse	Abcam	ab110413
Anti NIPSNAP1	Rabbit	Abcam	ab67302
Anti ODC (G-10)	Mouse	Santa Cruz	sc-390366
Anti AGO2	Mouse	Abcam	ab57113
Anti SEC16A	Rabbit	Proteintech	20025-1-AP
Normal IgG	Mouse	Santa Cruz	sc-2025
Secondary antibodies:			
Anti-mouse HRP-conjugated IgG		R&D Systems	HAF007
Anti-rabbit HRP-conjugated IgG		R&D Systems	HAF008
Anti-rat HRP-conjugated IgG		R&D Systems	HAF005
Anti-guinea pig DyLight Cy5		Jackson ImmunoResearch	706-495-148
Anti-mouse AlexaFluor 488		Jackson ImmunoResearch	715-545-150
Anti-mouse AlexaFluor 594		Jackson ImmunoResearch	715-585-150
Anti-rabbit Alexa Fluor 594		Jackson ImmunoResearch	711-585-152
Anti-goat Alexa Fluor 647		Jackson ImmunoResearch	705-605-003

Supplementary Table 3: List of primers

Target	Sequence
Hs mt-D Loop	Fwd: CTTCTGGCCACAGCACTTAAAC<br>Rev: GCTGGTGTTAGGGTTCTTTGTTTT
Hs mt-ND1	Fwd: CCACCTCTAGCCTAGCCGTTTA<br>Rev: GGGTCATGATGGCAGGAGTAAT
Hs mt-ND6	Fwd: CAAACAATGTTCAACCAGTAACCACTAC<br>Rev: ATATACTACAGCGATGGCTATTGAGGA
Hs mt-CYTB	Fwd: ATCACTCGAGACGTAAATTATGGCT<br>Rev: TGAACTAGGTCTGTCCCAATGTATG
Hs mt-CO1	Fwd: GACGTAGACACACGAGCATATTTCA<br>Rev: AGGACATAGTGGAAGTGAGCTACAAC
Hs mt-ATP6	Fwd: TAGCCATACACAACACTAAAGGACGA<br>Rev: GGGCATTTTTAATCTTAGAGCGAAA
Hs mt-RNR1	Fwd: TAGAGGAGCCTGTTCTGTAATCGAT<br>Rev: CGACCCTTAAGTTTCATAAGGGCTA
Hs ß$_2$M	Fwd: GCTGGGTAGCTCTAAACAATGTATTCA<br>Rev: CCATGTACTAACAAATGTCTAAAATGGT
