# Supplementary material for: Aneuploidy-induced proteostasis disruption impairs mitochondrial functions and mediates aggregation of mitochondrial precursor proteins through SQSTM1/p62
Source: Nat Commun. 2025 Jun 17;16:5328. doi: 10.1038/s41467-025-60857-4 (PMC12174339; doi:10.1038/s41467-025-60857-4)
Supplement: Supplementary file 2 — Description of Additional Supplementary Files [file 41467_2025_60857_MOESM2_ESM.pdf]

Description of Additional Supplementary Files

Supplementary Data 1: Statistical results of the global p62 proximity biotinylation data analysis including processed data, adjusted WT pair-comparisons and cellular compartment over-representations.

Supplementary Data 2: Statistical results of the autophagosome lumen p62 proximity biotinylation data analysis including processed data, adjusted WT and p62 cargo pair-comparisons, and cellular compartment over-representations.

Supplementary Data 3: Statistical results of the co-IP MS data analysis including processed data, IgG control pair-comparisons and p62 interactor cellular compartment over-representations.
