# Supplementary material for: Aneuploidy-induced proteostasis disruption impairs mitochondrial functions and mediates aggregation of mitochondrial precursor proteins through SQSTM1/p62
Source: Nat Commun. 2025 Jun 17;16:5328. doi: 10.1038/s41467-025-60857-4 (PMC12174339; doi:10.1038/s41467-025-60857-4)
Supplement: Supplementary file 6 — Reporting Summary [file 41467_2025_60857_MOESM6_ESM.pdf]

nature portfolio

Corresponding author(s): Prince Saforo Amponsah, Zuzana Storchova

Last updated by author(s): May 12, 2025

Reporting Summary

Nature Portfolio wishes to improve the reproducibility of the work that we publish. This form provides structure for consistency and transparency in reporting. For further information on Nature Portfolio policies, see our Editorial Policies and the Editorial Policy Checklist.

Statistics

For all statistical analyses, confirm that the following items are present in the figure legend, table legend, main text, or Methods section.

n/a	Confirmed	
☐	☒	The exact sample size (*n*) for each experimental group/condition, given as a discrete number and unit of measurement
☐	☒	A statement on whether measurements were taken from distinct samples or whether the same sample was measured repeatedly
☐	☒	The statistical test(s) used AND whether they are one- or two-sided<br>*Only common tests should be described solely by name; describe more complex techniques in the Methods section.*
☒	☐	A description of all covariates tested
☐	☒	A description of any assumptions or corrections, such as tests of normality and adjustment for multiple comparisons
☐	☒	A full description of the statistical parameters including central tendency (e.g. means) or other basic estimates (e.g. regression coefficient) AND variation (e.g. standard deviation) or associated estimates of uncertainty (e.g. confidence intervals)
☐	☒	For null hypothesis testing, the test statistic (e.g. *F*, *t*, *r*) with confidence intervals, effect sizes, degrees of freedom and *P* value noted<br>*Give P values as exact values whenever suitable.*
☒	☐	For Bayesian analysis, information on the choice of priors and Markov chain Monte Carlo settings
☐	☒	For hierarchical and complex designs, identification of the appropriate level for tests and full reporting of outcomes
☐	☒	Estimates of effect sizes (e.g. Cohen's *d*, Pearson's *r*), indicating how they were calculated

Our web collection on statistics for biologists contains articles on many of the points above.

Software and code

Policy information about availability of computer code

Data collection	Mass spectrometry data were obtained with Q Exactive mass spectrometer (Thermo Fisher Scientific).<br>Immunofluorescence images were captured using AxioObserver Z1 equipped with CSU-X1 spinning disk confocal head (Yokogawa) and Laser stack launch (3I, Denver, CO), and Nikon-confocal CSU-W1 SORA (2021) spinning disc microscope equipped with sCMOS cameras (MXR10015).<br>Western blots were imaged using Azure c500.<br>Attune NxT Flow Cytometer (Thermo Fisher Scientific) used for flow cytometry.<br>Promega Glomax Explorer microplate reader for measuring fluorescence (CellROX assays).
Data analysis	GraphPad Prism 9, Microsoft Excel, MaxQuant (version 2.0.1.0), R programming language (version 4.3.3; R Core Team 2024), R package ggplot2 (version 3.5.0), R package ggpubr (version 0.6.0), R package ggh4x (version 0.2.8), R package cowplot (version 1.1.3), R package idpr (version 1.12.0), WebGestalt R package (version 0.4.6), R package pcaMethods (version 1.94.0), R package limma (version 3.58.1), Slidebook 6, Nikon NIS Elements AR software (version 5.30.06), FlowJo software (version 10), Image J, EzColocalization plugin for Image J, Mitochondria Analyzer plugin for Image J

For manuscripts utilizing custom algorithms or software that are central to the research but not yet described in published literature, software must be made available to editors and reviewers. We strongly encourage code deposition in a community repository (e.g. GitHub). See the Nature Portfolio guidelines for submitting code & software for further information.

Data

Policy information about availability of data

All manuscripts must include a data availability statement. This statement should provide the following information, where applicable:

- Accession codes, unique identifiers, or web links for publicly available datasets
- A description of any restrictions on data availability
- For clinical datasets or third party data, please ensure that the statement adheres to our policy

The mass spectrometry data have been deposited to the ProteomeXchange Consortium via the PRIDE partner repository with the dataset identifiers PXD052623, PXD061687, PXD061712, PXD052174, PXD061691 and PXD052637. Normalized data are available in Supplementary Data 1-3.
Multi-omics data of CCLE cancer cell lines, including aneuploidy scores, RNAseq transcriptomics, mass spectrometry proteomics and gene dependency data were downloaded through the DepMap data portal (version 22Q4). Gene Ontology Cellular Compartments (GOCC) and other gene sets were downloaded from the Molecular Signature database (version 2022.1). Information on macromolecular complexes were downloaded from the CORUM database. A list of 148 reported p62 autophagosomal lumen cargo proteins were taken from the supplementary data of Zellner et al. 2021

Research involving human participants, their data, or biological material

Policy information about studies with human participants or human data. See also policy information about sex, gender (identity/presentation), and sexual orientation and race, ethnicity and racism.

Reporting on sex and gender	n/a
Reporting on race, ethnicity, or other socially relevant groupings	n/a
Population characteristics	n/a
Recruitment	n/a
Ethics oversight	n/a

Note that full information on the approval of the study protocol must also be provided in the manuscript.

Field-specific reporting

Please select the one below that is the best fit for your research. If you are not sure, read the appropriate sections before making your selection.

☒ Life sciences ☐ Behavioural & social sciences ☐ Ecological, evolutionary & environmental sciences

For a reference copy of the document with all sections, see nature.com/documents/nr-reporting-summary-flat.pdf

Life sciences study design

All studies must disclose on these points even when the disclosure is negative.

Sample size	A minimum of 3 biological replicates, unless indicated in the figure legend, were performed. N is specified in figure legends.
Data exclusions	No data were excluded from the analysis
Replication	All the experiments were performed from at least three independent replicates (unless stated otherwise), the reproducibility was statistically evaluated, the applied statistical tests are noted in figure legends or in material and methods.
Randomization	n/a
Blinding	n/a

Reporting for specific materials, systems and methods

We require information from authors about some types of materials, experimental systems and methods used in many studies. Here, indicate whether each material, system or method listed is relevant to your study. If you are not sure if a list item applies to your research, read the appropriate section before selecting a response.

Materials & experimental systems

n/a	Involved in the study
☐	☒ Antibodies
☐	☒ Eukaryotic cell lines
☒	☐ Palaeontology and archaeology
☒	☐ Animals and other organisms
☒	☐ Clinical data
☒	☐ Dual use research of concern
☒	☐ Plants

Methods

n/a	Involved in the study
☒	☐ ChIP-seq
☐	☒ Flow cytometry
☒	☐ MRI-based neuroimaging

Antibodies

Antibodies used

Mouse anti p62 (SQSTM-1) lck ligand, BD transduction, # 610833/610832
Guinea pig anti p62/ SQSTM1 (C-terminus), Progen, # GP62-C
Mouse anti p62, Santa Cruz, # sc-28359
Rabbit anti LC3B, Abcam, # ab48394
Rabbit anti BIOTIN, Abcam, # ab1227
Goat anti c-MYC, Bethyl Labs, # A190-104A
Mouse anti LAMP1 [H4A3], Abcam, # ab25630
Mouse anti KEAP1, Santa Cruz, # sc-365626
Mouse anti TAX1BP1, Santa Cruz, # sc-393143
Mouse anti NBR1, Santa Cruz, # sc-130380
Mouse anti CTSZ, Santa Cruz, # sc-376976
Mouse anti PELO, Santa Cruz, # sc-393418
Mouse anti HSP27, Enzo Life Sciences, # ADI-SPA-800-D
Rabbit anti HSF1, Santa Cruz, # sc-9144
Rabbit anti FLAG, Sigma-Aldrich, # F7425
Mouse anti GFP, Santa Cruz, # sc-9996
Rat anti GFP, Chromotek, # 3h9-100
Rabbit anti IMMT/Mitofilin, Biomol, # A305-023A-M
Mouse anti MRPL45 (E-12), Santa Cruz, # sc-515563
Rabbit anti TOMM20 [EPR15581-54], Abcam, # ab186735
Mouse anti HADHA (E-8), Santa Cruz, # sc-374497
Mouse anti HADHB (E-1), Santa Cruz, # sc-271495
Mouse Total OXPHOS Rodent WB Antibody Cocktail, Abcam, # ab110413
Rabbit anti NIPSNAP1, Abcam, # ab67302
Mouse anti ODC (G-10), Santa Cruz, # sc-390366
Mouse anti AGO2, Abcam, # ab57113
Rabbit SEC16A, Proteintech, # 20025-1-AP
Normal Mouse IgG, Santa Cruz, # sc-2025
Anti-mouse HRP-conjugated IgG, R&D Systems, #HAF007
Anti-rabbit HRP-conjugated IgG, R&D Systems, #HAF008
Anti-rat HRP-conjugated IgG, R&D Systems, #HAF005
Anti-guinea pig DyLight Cy5, Jackson ImmunoResearch, #706-495-148
Anti-mouse AlexaFluor 488, Jackson ImmunoResearch, #715-545-150
Anti-mouse AlexaFluor 594, Jackson ImmunoResearch, #715-585-150
Anti-rabbit Alexa Fluor 594, Jackson ImmunoResearch, #711-585-152
Anti-goat Alexa Fluor 647, Jackson ImmunoResearch, #705-605-003
Supplementary table 2 lists all antibodies used in this study.

Validation

All commercially available antibodies were used according to the validations performed by the manufacturers.

Eukaryotic cell lines

Policy information about cell lines and Sex and Gender in Research

Cell line source(s)

Parental HCT116 (45, X) were purchased from ATCC® (CCL-247)

Authentication

Sequencing and karyotyping of parental and derived polysomic cells were previously performed as described in Stingele et al. 2012, Kneissig et al. 2019 (referenced) and confirmed for this study

Mycoplasma contamination

All the cell lines are periodically tested for mycoplasma contamination using PlasmoTest (InvivoGen).

Commonly misidentified lines (See ICLAC register)

No commonly misidentified cell lines were used in this study.

Plants

Seed stocks	n/a
Novel plant genotypes	n/a
Authentication	n/a

Flow Cytometry

Plots

Confirm that:

☒ The axis labels state the marker and fluorochrome used (e.g. CD4-FITC).

☒ The axis scales are clearly visible. Include numbers along axes only for bottom left plot of group (a 'group' is an analysis of identical markers).

☐ All plots are contour plots with outliers or pseudocolor plots.

☒ A numerical value for number of cells or percentage (with statistics) is provided.

Methodology

Sample preparation	Cultured cells were stained simultaneously with MitoTracker Green FM (Invitrogen, #M7514) and Deep Red FM (Invitrogen, #M22426) dyes according to the manufacturer's instructions to analyze mitochondria mass and membrane potential, respectively, and incubated for 20 min at 37 °C and 5% CO2. Unstained cells were included for background control. After incubation, cells were washed five times with PBS before harvesting by trypsinization. Cells were then resuspended in PBS and analyzed on an Attune NxT acoustic focusing cytometer (Thermo Fisher). The gating, data analysis, and visualization were performed using the FlowJo software (version 10).
Instrument	Attune NxT acoustic focusing flow cytometer
Software	Attune NxT Software 3.1.1243.0
Cell population abundance	Total cells: > 80%, Single cells: > 90%
Gating strategy	Singlets were gated from a starting cell population of 50,000 using SSC-A/FSC-A. A histogram was plotted from this population and cells with background staining excluded using the unstained background control population

☒ Tick this box to confirm that a figure exemplifying the gating strategy is provided in the Supplementary Information.
